# Molecular Biological Research on the Pathogenic Mechanism of Retinoblastoma

**DOI:** 10.3390/cimb46060317

**Published:** 2024-05-27

**Authors:** Xiangyi Ma, Xinyu Li, Qi Sun, Fuxiao Luan, Jing Feng

**Affiliations:** Department of Ophthalmology, Beijing Chaoyang Hospital, Capital Medical University, Beijing 100020, China; symxy@mail.ccmu.edu.cn (X.M.); lxywdy@gmail.com (X.L.); sunqiamm@163.com (Q.S.)

**Keywords:** retinoblastoma, molecular biological, *RB1*, epigenetics

## Abstract

Retinoblastoma (RB) is the most common intraocular malignant tumor in children, primarily attributed to the bi-allelic loss of the *RB1* gene in the developing retina. Despite significant progress in understanding the basic pathogenesis of RB, comprehensively unravelling the intricate network of genetics and epigenetics underlying RB tumorigenesis remains a major challenge. Conventional clinical treatment options are limited, and despite the continuous identification of genetic loci associated with cancer pathogenesis, the development of targeted therapies lags behind. This review focuses on the reported genomic and epigenomic alterations in retinoblastoma, summarizing potential therapeutic targets for RB and providing insights for research into targeted therapies.

## 1. Introduction

Retinoblastoma (RB) is the most common intraocular malignant tumor in children with an incidence of 1:16,000 live births, which originates from the mature precursor of the cone in the developing retina [1]. RB damages a patient’s sight, appearance, and mental health, and metastasis can even be life-threatening [2]. The survival rate of retinoblastoma in developed countries is over 95% [3,4]; however, in low-income countries, survival rates are significantly lower. In Uganda, the five-year survival rate is 60%, while in Senegal, it is 53% [5,6]. In a study encompassing 13 countries across six continents, the rate of eye removal was 48.1% [7]. Currently, clinical treatment modalities for RB are limited, mainly including laser therapy, cryotherapy, and radiotherapy, as well as systemic chemotherapy and enucleation to remove the entire tumor. In addition, the injection of drugs into the vitreous body can also be used, with melphalan and topotecan being the most commonly utilized. Nevertheless, potential side effects such as vitreous hemorrhage, secondary uveitis, and secondary glaucoma may manifest [8,9]. Therefore, there is a need to transition from conventional treatment approaches to targeted therapies to reduce the risk of complications and achieve precise, efficient treatment. This article reviews the molecular biology pathogenesis of RB, offering insights for research into targeted therapies for the condition.

## 2. Clinical Genetics of Retinoblastoma

RB is thought to be related to the mutation or inactivation of two copies of the retinoblastoma gene (*RB1*), the tumor suppressor gene, and caused by a mutation of *RB1* programming retinoblastoma protein (pRB) [10]. RB can be hereditary or non-hereditary, depending on the heredity.

### 2.1. Hereditary Retinoblastoma

**Figure 1:**
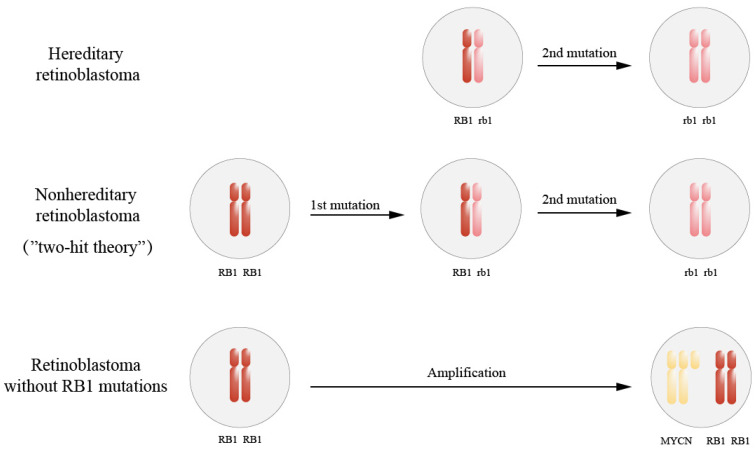
Genetics of retinoblastoma. In hereditary retinoblastoma, all cells in the body lack one of the functional copies of *RB1*, and tumors occur where the remaining copy is lost. In nonhereditary retinoblastoma, noncancerous cells show no defect in either copy of *RB1*. Therefore, they require two hits in a single retinal cell lineage to inactivate both copies of the *RB1* gene. A small percentage of patients have no mutations in the *RB1* gene, some of whom will develop the disease through the amplification of the MYCN gene.

Approximately 40% of RB is congenital (hereditary) (Figure 1) comprising bilateral and multifocal diseases [11], and is generally observable by 12 months of age [12]. Besides retinal cells, all somatic cells of patients are *RB1*-gene-mutated [13]. Hereditary retinoblastoma is autosomal-dominant, mainly resulting from pathogenic mutation, and is bilateral RB (about 20–30%), or unilateral and multiple RB (about 70–80%). When accompanied by intracranial tumors, such as pineal body tumors and primary retinoblastoma or nearby, it is called trilateral RB [14]. Survivors of hereditary RB, especially those who are treated using external irradiation radiotherapy, have a significantly increased risk of developing malignant tumors [15].

### 2.2. Non-Hereditary Retinoblastoma

Approximately 60% of RBs are acquired (non-hereditary), and the genetic mutation is simply due to the acquired mutation of retinal cells [11]. The *RB1* gene of genital cells and cells of other tissue and organs remain virtually normal, with unilateral episodes usually being observed by 24 months of age [12]. A small percentage of non-hereditary retinoblastomas are caused by MYCN amplification and normal *RB1*.

## 3. Retinoblastoma Genomics

The pathogenesis of retinoblastoma involves abnormalities in multiple genes. The bi-allelic inactivation of the *RB1* gene is a major driving force in RB development, referred to as the “two-hit theory”, involving two successive mutations [16]. Additionally, RB patients typically exhibit mutations in other oncogenes, further promoting tumor formation.

### 3.1. Two Alleles of the RB1 Gene Inactivated

The “two-hit theory” indicates that the inactivation of two alleles of the tumor suppressor gene is necessary for cancer, leading to uncontrolled cell differentiation and tumor formation. Other genes rarely change except for *RB1* inactivation, which is correlated to a differentiated tumor-expressing mature cone marker, called RB subtype 1 [17]. The *RB1* gene is the first recorded tumor suppressor gene, located on chromosome 13q14.2, spanning about 180 Kb and including 27 exons, programming the nucleophosphoprotein called pRB [18,19]. pRB, a kind of chromosome-associated protein, regulates the transcription of important genes, including cell cycle genes and DNA-damage-repairing genes. The phosphorylation of pRB removes its suppression of E2F during the G1/S transforming process, which promotes cell cycle progress [20].

In the mouse model, deleting *RB1* induces widespread new retinal vessel growth, with autophagy and the dissolution of photoreceptors. More specifically, *RB1* directly binds to the particular HIF target gene and restrains its expression [21]. Structurally, RB is a multidomain protein with an N-terminal domain and A and B pockets, which together form the small pocket structure, along with a C-terminal domain [22]. RB interacts with more than 200 different types of proteins, making RB the hub of a broad interaction network. Mayra Martínez-Sánchez summarized the interaction of RB with chromosome-modifying factors, including DNMT1, HDAC 1, 2, and 3, SIRT1, replication factor C, DNA polymerase alpha, MDMX, and MDM2 [23]. The renowned p53 regulator MDM2 exhibits a dual role in regulating RB activity: during the G1 phase, it can recognize and bind to RB mRNA, facilitating RB translation by transporting it to the polysomes; however, during G2, MDM2 recognizes and degrades the RB protein. This dual functionality underscores the significance of MDM2 as a pivotal regulator of RB activity [24].

There are some conflicting opinions about the “two-hit theory”. Domenico Mastrangelo et al. argued that it is an outdated view of cancer etiology because the theory does not take into account a large number of new acquisitions, such as chromosomal and epigenetic variations. The authors demonstrated that the clinical and epidemiological data reported to date do not fully satisfy the predictions of the “two-hit theory” [25,26].

### 3.2. Associated Oncogene Mutation

In recent years, many studies have shown that changes in RB genes, other than those causing RB gene inactivation, are common in RB patients and promote tumor progression, also known as subtype 2 [17].

#### 3.2.1. Genes Related to Cell Division and Proliferation

MYCN is the most common amplified gene on 2p. The amplification and expression of MYCN are present in *RB1*-mutated RBs and can also be independent of *RB1* mutations [27], usually hypermethylated [28,29]. MYCN-amplified RBs express less differentiated cone markers, as well as neuronal/ganglion cell markers with significant inter- and intra-tumor heterogeneity, and this type of cone dedifferentiation is associated with stemness. MYCN plays a central role in regulating a range of cellular functions that drive carcinogenesis, including cell cycle progression, cell growth, proliferation, and apoptosis. Targeted MYCN knockout inhibits cell growth, induces cell cycle arrest, and promotes apoptosis in RB cells [30]. However, despite MYCN amplification in RB, its amplification is not associated with any histological high-risk factors, such as optic nerve or choroidal invasion [31].

The most commonly amplified genes on 6p include the DNA-binding oncogene DEK and the transcription factor E2F3 [32]. DEK is involved in chromatin remodeling and gene transcription, playing a crucial role in cellular apoptosis [33]. E2F3, a member of the E2F transcription factor family, is considered a transcriptional activator, is regulated by the *RB1* protein, and is frequently overexpressed in cancer. Enhanced DEK expression could endow cancer cells with stem cell-like characteristics, fostering tumor advancement and resistance to chemotherapy, underscoring its pivotal role as an oncogenic driver in tumor initiation [34].

Meng Chen et al. found that the E2F1/CKS2/PTEN axis is involved in regulating the malignant phenotype of RB, and cyclin-dependent kinase regulatory subunit 2 (CKS2) is a new regulatory factor that regulates tumor-related phenotypes in RB. The expression of CKS2 is abnormally high in RB. In the Y79 retinoblastoma cell line, the deletion of CKS2 resulted in reduced cell proliferation, delayed DNA replication, and reduced clone growth. Downregulating CKS2 also slowed the growth of tumor xenografts in nude mice. The transcription factor E2F1 enhances the expression of CKS2 by binding to its promoter, and CKS2 regulates the PI3K/AKT pathway associated with cancer [35].

Ubiquitin E2 ubiquitin-conjugating enzyme 2T (UBE2T) is a member of the ubiquitin-conjugating enzyme family, which interacts with FANCL to ubiquitinate FANCD2 when replication forks are stalled by DNA damage [36]. UBE2T significantly participates in the proliferation of RB through the STAT3 signaling pathway [37]. The upregulation of UBE2T is associated with increased infiltration of Th2 cells, while its downregulation of UBE2T can induce cell cycle arrest in the G2/M phase and reduce the proliferation of RB cells [38].

#### 3.2.2. Genes Related to Infiltration and Invasion

CDH11, the gene encoding the adhesion protein cadherin-11, is commonly deleted on chromosome 16q [39]. CDH11 is a classical cell adhesion glycoprotein that mediates cell–cell adhesion and acts as a tumor suppressor in RB [40]. Nikia Laurie et al. found that the loss of CDH11 in retinoblastoma cells may lead to tumor progression and invasion of the optic nerve [41].

The expression level of claudin-1 is negatively correlated with RB cell differentiation, optic nerve invasion, and clinical stage [42]. Meanwhile, claudin-1 binds with other claudins to form tight junctions, plays an important role in epithelial barrier function, and is involved in the development of a variety of cancers [43]. In RB tissue, claudin-1 expression is low, and the integrity of the connections between cells may be lost.

In contrast to claudin-1, meta-analysis suggests that MMP-1, MMP-2, MMP-9, and VEGF are highly expressed in RB, which are highly correlated with poor cell tumor differentiation, tumor invasion, and clinically advanced stage [44]. Placental growth factor (PlGF) is a member of the vascular endothelial growth factor (VEGF) family, which induces endothelial cell proliferation and migration, as well as anti-endothelial cell apoptosis, increases the expression of vascular permeability, and is often involved in tumor angiogenesis. PlGF overexpression promotes the growth and metastasis of RB tumor cells.

Centromere protein E (CENPE) is highly expressed in human tumors, and its mRNA and protein levels in retinoblastoma cells are also significantly upregulated. The expression of CENCE is associated with the invasive behavior of retinoblastoma, and its mechanism may involve immune infiltration, as well as interactions with non-coding RNAs and transcription factors [45]. The interaction of these factors induces RB invasion and promotes tumor cell progression.

The BCL6 corepressor (BCOR) is a transcription factor that acts as a tumor suppressor gene in hematopoiesis, embryogenesis, and lymphoid development [46]. BCOR alterations were found to be present in 72.7% of cases. Francis et al. also found that among 83 RB patients, 22.9% had BCOR mutations, which are associated with poor prognosis and specific metastasis-free survival duration [47]. The estrogen-related receptor gamma (ESRRG) is a crucial mediator of RB hypoxia adaptation and cell survival, activated by *RB1* deficiency. Independently of *RB1*, BCOR inhibits ESRRG-mediated transcription. Therefore, BCOR deficiency enables retinoblastoma cells to survive under hypoxic conditions [48].

## 4. Epigenetics of RB

Epigenetics refers to heritable changes in gene expression that occur without alterations in the DNA sequence. These changes primarily include DNA methylation, histone modifications, and the expression of non-coding RNAs. Aberrations in epigenetics can lead to the activation of oncogenes and the silencing of tumor suppressor and DNA repair genes, thereby initiating and promoting tumorigenesis. Due to its reversibility, epigenetic regulation offers new ways for more effective treatment methods.

### 4.1. DNA Methylation

#### 4.1.1. Pathogenesis Related to DNA Methylation

It has been reported that individuals exhibiting heightened methylation levels across their genome are at increased risk of RB [49]. Greger et al. initially demonstrated the methylation of the CpG island (CpG 106) of the *RB1* promoter in 1989 [50], which leads to gene silencing and reduced expression of associated genes. Subsequent research has continually identified the methylation of the *RB1* promoter [51], underscoring the epigenetic impact on RB progression.

lncRNA MEG3 exhibits tumor-suppressive properties, inhibiting tumorigenesis. In RB, the elevated expression of DNA methyltransferase 1 (DNMT1) prompts MEG3 promoter methylation, consequently suppressing MEG3 expression. This alteration influences the Wnt/β-catenin pathway, promoting cell proliferation [52,53]. A similar mechanism has been observed in diabetic retinopathy, where decreased MEG3 expression facilitates endothelial-mesenchymal transition (endMT) via the PI3K/Akt/mTOR signaling pathway [54], potentially implicating its role in RB as well. Conversely, lncRNA can affect DNA methylation by recruiting DNMT to specific sites [54], and both interact and influence each other in the pathogenesis of RB.

#### 4.1.2. Diagnosis and Treatment

Researchers have observed elevated methylation levels of TFAP2A, a transcription factor, in the aqueous humor of RB patients compared to healthy individuals, suggesting its potential as a diagnostic marker for RB [55]. This is consistent with similar observations in melanoma [56]. Furthermore, the promoter of transcription factor Pax5 has high methylation levels in Rb, resulting in the downregulation of Pax5 expression. An anti-tumor drug known as Cyclophosphamide has shown potential in preventing Pax5 methylation. The increase in Pax5 expression leads to the inhibition of RB proliferation by modulating the Notch1 signaling pathway [57].

Additionally, numerous potential targets for RB treatment have been identified in a recent study, including 193 differentially expressed genes and 74 differentially methylated genes [58]. These findings underscore the substantial role of DNA methylation in RB pathogenesis, elucidating disease mechanisms and offering potential ways for therapeutic intervention.

### 4.2. Histone Modification

#### 4.2.1. Pathogenesis Related to Histone Modification

In the pathogenesis of RB, histone modifications have emerged as influential regulators of key processes, shaping the epigenetic profile that influences disease progression. Vascular Endothelial Growth Factor A (VEGFA) is known for its involvement in processes such as angiogenesis, cell proliferation, and blood vessel permeability. Utilizing the GEO database, it has revealed that there is an association between VEGFA expression and RB. Further research showed that the methylation modification of the histone H3K4me3 on the VEGFA promoter correlates with increased VEGFA expression, fostering RB development [59].

The epigenetic impact extends to lncRNAs, significantly influencing the progression of RB. LincRNA-ROR plays a pivotal role in tumor invasiveness and metastasis. In RB, H3K27 acetylation at the lincRNA-ROR promoter promotes its expression, subsequently facilitating the epithelial-mesenchymal transition (EMT) program and Notch signaling pathway, propelling tumor progression [60,61]. The upregulation of histone acetylation at chr12p13.32, involving modifications like H3K4me, H3K4me3, H3K9ac, and H3K27ac, activates lncRNA GAU1 and oncogene GALNT8, instrumental in propelling RB progression [62].

#### 4.2.2. Treatment

A nonclassical oncogene, PI3Kγ, emerges as a pivotal player activated across various malignancies [63]. LncRNA CANT1 hinders histone H3K4 trimethylation at the promoter region of PI3Kγ, effectively inhibiting its expression and the progression of RB [64]. In other malignant tumors, CANT1 is also involved in regulating tumor progression by controlling the transcription of key genes. In uveal melanoma (UM), CANT1 regulates the transcription of essential tumor suppressors, JPX and FTX, by promoting H3K4 methylation in the promoters [65]. These studies suggest that CANT1 may be a potential common target for the treatment of various cancers.

The oncogene EZH2 employs H3K27 trimethylation to induce gene silencing, contributing to RB pathogenesis [66]. Interestingly, miR-101-3p engages with EZH2, impeding RB tumor cell formation [67], while the inhibition of EZH2 through specific inhibitors displays therapeutic potential against RB by inhibiting proliferation and metastasis [68].

Due to the impact of histone acetylation on RB development, it is possible to modulate RB progression by targeting acetylation-related processes. As an inhibitor of histone deacetylase 6 (HDAC6), WT161 exerts anti-tumor effects by enhancing the acetylation of histones H3 and H4 on the promoter of the apoptosis-related gene BAD, prompting cell apoptosis and evoking anti-tumor responses [69].

### 4.3. Non-Coding RNA (ncRNA) Regulation

In RB, miRNAs can recognize target mRNA through complementary base pairing, subsequently leading to the degradation of target mRNA or the inhibition of its translation (Table 1). Many lncRNAs exert a unique influence by serving as miRNA sponges, thereby modulating miRNA functions (Table 2). Due to the significant role of miRNAs in gene expression regulation, the capacity of lncRNAs to sponge miRNAs introduces an additional layer of complexity to the regulatory network, ultimately shaping the progression of RB.

**Figure 2:**
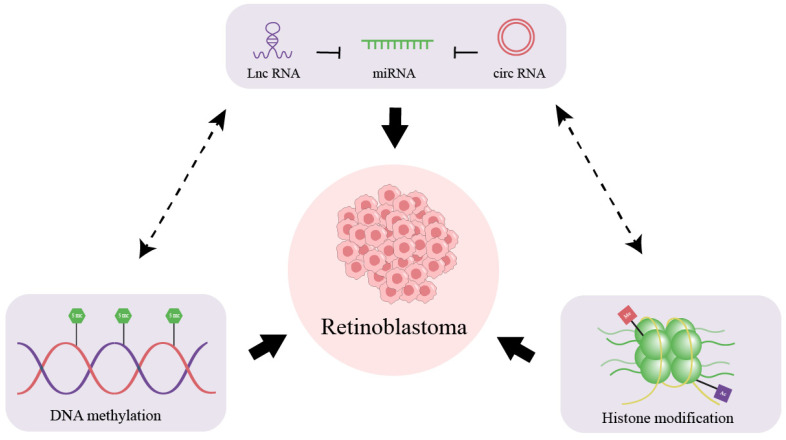
Schematic illustration of epigenetics of RB. The interplay among DNA methylation, histone modification, and non-coding RNA expression mutually influences the pathogenesis of RB, collectively contributing to its development. LncRNAs and circRNAs can act as molecular sponges for miRNAs, inhibiting their function.

The prominent function of circRNAs lies in their ability to act as molecular sponges for miRNAs, thus impeding their regulatory activity (Table 3). The ability to modulate critical cellular processes and interact with key signaling pathways makes circRNAs promising candidates for innovative therapeutic interventions aimed at mitigating RB progression. This interplay between lncRNAs, circRNAs, and miRNAs offers novel insights into understanding and potentially intervening in the pathophysiology of RB (Figure 2).

In addition to being used for exploring the regulatory mechanisms of non-coding RNAs, the WERI-RB1 and similar cell lines also play a crucial role in studying chemotherapy resistance.

Chemotherapy before and after enucleation is one of the most common treatment methods for RB. The DNA alkylating agent carboplatin and the topoisomerase inhibitor etoposide are commonly used to treat RB, and their combined application often yields better results [95]. Topoisomerases are essential enzymes involved in DNA relaxation for transcription and replication, DNA repair, and chromatin remodeling. Etoposide acts by inhibiting these enzymes, resulting in DNA cleavage and, thus, exerting an anti-tumor effect [96,97]. However, the long-term and widespread use of chemotherapy drugs can lead to resistance, promoting tumor invasion and metastasis. Therefore, delaying the emergence of chemoresistance remains a priority in treatment.

Chemoresistance in RB has been investigated in various cell culture models. The WERI-RB1 cell line, established from spontaneously grown enucleated RB eyes [98], has been utilized to determine the mechanisms of chemoresistance. By increasing the dosage of etoposide and harvesting the surviving cells, the etoposide-resistant subclone WERI-ETOR was established [99].

The transient receptor potential melastatin 8 (TRPM8) channel, a member of the TRP superfamily, plays a role in etoposide-sensitive cancer types, participating in intracellular calcium regulation in RB [100]. Ca^2+^, as a trigger and regulator in the apoptotic death process, modulates cell apoptosis and is closely associated with cancer progression [101]. Nerve growth factor (NGF), a neurotrophic factor, is nearly four times higher in RB patients compared to controls [102]. It has been reported that NGF is involved in the regulation of TRPM8 activation, thereby controlling the influx of Ca^2+^, influencing the sensitivity of RB cells to etoposide [103]. Additionally, Vinodh Kakkassery et al. determined that the pathways “retinoid metabolism and transport” and “sphingolipid de novo biosynthesis” are associated with etoposide resistance [104]. Furthermore, Jacqueline Reinhard et al. demonstrated that components of the extracellular matrix play a crucial role in the development of chemotherapy resistance in RB [105].

## 5. Pathology of RB

Retinoblastoma consists of hyperchromatic cells with a high ratio of nucleus to cytoplasm [106]. Depending on the degree of differentiation, tumors can be classified as well differentiated (Homer Wright (HW) rosettes) or poorly differentiated (Flexner-Wintersteiner rosettes) [107]. These pathologic phenotypes reflect the degree of tumor differentiation and are also key factors in the prediction of the tumor grade and prognosis of RB.

In the center of HW rosettes, the absence of a distinct lumen signifies a high degree of tumor differentiation, whereas FW rosettes indicate an early stage of retinal differentiation with a visible lumen at the center [107] (Table 4). The dual presence of two rosettes is recognized as a characteristic pathological feature of RB [28]. Recently, a third rosette has been discovered, exhibiting a larger size but sharing the same peripheral outer cells as HW and FW rosettes [108].

As the tumor invades the optic nerve or choroid tissue, the risk of metastasis and spread of the tumor increases. Specific characteristics in enucleated globes, such as retrolaminar optic nerve invasion or massive choroidal invasion, are acknowledged as high-risk factors linked to RB metastasis [109]. The identification of these risk factors aids in determining indications for adjuvant chemotherapy and reducing the risk of distant metastasis and local recurrence [106].

Yaqoob et al. found that among 54 patients, the main histopathological traits included choroidal invasion, observed in 18 patients, along with the anterior invasion of the optic nerve anterior to the lamina cribrosa, observed in 16 patients. Through association analysis, the researchers also found that clinical features such as pseudohypopyon, iris neovascularization, buphthalmos, and glaucoma were associated with choroidal infiltration [110]. Additionally, Kashyap et al. also highlighted correlations between features such as glaucoma and iris neovascularization and the incidence of high-risk histopathological findings [111].

Specific clinical features observed during patient visits can aid in identifying high-risk histopathological characteristics and predicting metastasis. The information is pivotal for selecting the most suitable treatment strategies and enhancing the survival rate of RB patients.

## 6. Conclusions

RB, as a prevalent intraocular malignancy in children, is regulated by multiple factors in its pathogenic mechanisms. Clinical genetic studies of RB have revealed the relationship between mutations or the inactivation of the *RB1* gene and hereditary RB, providing a clear foundation for understanding its pathogenesis of RB. On the other hand, non-hereditary RB has also been investigated, and mutations in relevant oncogenes have been closely associated with its development. Genomic research on RB has identified disruptions in signaling pathways and bi-allelic inactivation of the *RB1* gene as important mechanisms underlying RB initiation. Genomic studies provide us with a deeper understanding of the pathogenesis of RB, enabling more precise diagnosis and treatment of this disease.

Furthermore, epigenetic regulation involving non-coding RNAs, DNA methylation, and histone modifications also plays a significant role in the development of RB, with important implications for its diagnosis, treatment, and prognosis. The targeting of epigenetic modifications provides a unique opportunity to optimize therapeutic paradigms and develop novel treatment modalities. Epigenetic editing offers significant advantages in terms of safety, as it can regulate gene expression and protein levels without directly modifying DNA sequences, thereby avoiding the potential side effects associated with gene editing tools. This capability provides an additional safety measure for treating more common diseases. Another notable advantage of epigenetic editing is its ability to edit without being limited by specific types of gene mutations, significantly increasing the flexibility and applicability of treatment options. However, optimizing delivery systems remains a widespread and critical challenge. Ensuring that therapeutic agents can be effectively delivered to specific cells and tissues is key to successful treatment, and it often represents a major bottleneck in translating theory into clinical therapies. Despite the continual identification of epigenetic sites relevant to cancer therapy, many of these studies remain limited. The pathogenesis of RB involves the complex regulation of multiple genes and pathways; however, the respective roles and interrelationships between this large, complex RNA regulatory network and protein-based regulatory mechanisms have not been clearly understood. Furthermore, while some biomarkers and gene expression features associated with RB prognosis have been identified, there is currently a lack of reliable predictive models to guide clinical diagnosis. Future efforts should also entail further clinical trials of these targeted treatment approaches to demonstrate their safety and efficacy.

## Figures and Tables

**Table 1:** Role of miRNAs in RB.

miRNA	Target	Effects on Cell Proliferation, Migration, and Invasion	Type of Cell Line	Clinical Value
miR-142-5p [70]	PTEN	Promote	ARPE-19, WERI-RB1, Y79, SO-RB50, and HXO-RB44	TNM stage and tumor size
miR-181a-5p [71]	NRAS	Suppress	ARPE-19, HXO-RB44, SO-RB50, Y79, and WERI-RB1	Tumor aggressiveness, tumor size, and clinical stage
miR-98 [72]	IGF1R	Suppress	ARPE-19, WERI-RB1, Y79, and SO-RB50	Differentiation, N classification, and largest tumor base
miR-153-3p [73]	IGF1R	Suppress	ARPE-19, WERI-RB1, and Y79	Tumor base and differentiation
miR-214-3p [74]	ABCB1, XIAP	Suppress	ARPE-19, WERI-RB1, SO-RB50, and Y79	ICRB stage and chemotherapy resistance; favorable outcome in Kaplan–Meier analysis
miR-340 [75]	WIF1	Promote	ARPE-19, WERI-RB1, SO-RB50, and Y79	Tumor size, ICRB stage, and optic nerve invasion; worse overall survival in Kaplan-Meier analysis

**Table 2:** Role of lncRNAs in RB.

LncRNA	Target	Effects on Cell Proliferation, Migration, and Invasion	Type of Cell Line	Clinical Value
X–inactive specific transcript (XIST) [76]	miR-191-5p/BDNF	Promote	ARPE-19, HXO-RB44, Y79, WERI-RB1, and SO-RB50	Tumor size, choroidal nerve invasion, optic nerve invasion, and tumor staging
lncRNA-UCA1 [77]	PI3K/Akt pathway	Promote	ACBRI-181, HXO-RB44, and Y79	Tumor size, optic nerve invasion, and pathologic grade
HEIH [78]	miR-194-5p/WEE1	Promote	ARPE-19, Y79, and SO-RB50	TNM stage, optic nerve invasion, and choroidal invasion
LINC00324 [79]	miR-769-5p/STAT3	Promote	ARPE-19, Y79, SO-RB50, and WERI-RB1	TNM stage and optic nerve invasion
LINC00205 [80]	miR-665/HMGB1	Promote	ARPE-19, Y79, SO-RB50, and WERI-RB1	Differentiation grade, TNM stage, and optic nerve invasion
CASC9 [81]	miR-145-5p/E2F3.	Promote	ARPE-19, Y79, and WERI-RB1	Clinical stages, differentiation, and optic nerve invasion
LINC00115 [82]	miR-489-3p/PFKFB2	Promote	ARPE-19, Y79, SO-RB50, and HXO-RB44	Choroidal invasion, optic nerve invasion, and TNM stage
TMPO-AS1 [83]	TMPO-AS1/HIF-1α	Promote	HXO-RB44 and SO-RB50	Clinical stage
SNHG16 [84]	miR-182-5p, miR-128-3p/LASP1	Promote	ARPE-19, WERI-RB1, SO-RB50, and Y79	TNM stage, choroidal and optic nerve invasion; poor overall survival time in Kaplan-Meier survival analysis
SND1-IT1 [85]	miR-132-3p/SMAD2	Promote	ARPE-19, Y79, SO-RB50, and WERI-RB1	Tumor size, choroidal invasion, and optic nerve invasion; shorter overall survival time in Kaplan-Meier survival analysis
FEZF1-AS1 [86]	miR-363-3p/PAX6 Axis	Promote	ARPE-19, WERI-RB1, and Y79	Less survival time in Kaplan-Meier survival analysis
KCNQ1OT1 [87]	miR-134/TRIM44	Promote	ARPE-19, Weri-RB1, and Y79	Shorter disease-free survival time
TP53TG1 [88]	miR-33b/SHCBP1	Promote	ARPE-19, SO-RB50, WERI-RB1, Y79, and RBL-13	Shorter overall survival time in Kaplan-Meier survival analysis
LEF1-AS1 [89]	Wnt/β-catenin pathway	Promote	ARPE-19, SO-RB50, and HXO-RB44	Shorter disease-free survival time
ZFPM2-AS1 [90]	miR-515/HOXA1	Promote	ARPE-19, WERI-RB1, SO-RB50, and Y79	Dismal prognosis

**Table 3:** Role of circRNAs in RB.

circRNA	Target	Effects on Cell Proliferation, Migration, and Invasion	Type of Cell Line	Clinical Value
hsa_circ_0001649 [91]	AKT/mTOR	Suppress	ARPE-19, Y79, SO-RB50, and HXO-RB44	Tumor size and IIRC stage
circ_0000527 [92]	miR-646/BCL-2	Promote	ARPE-19, Y79, HXO-RB44, SO-RB50, and WERI-RB1	Tumor size, optic nerve invasion, and tumor stage
circ_0000034 [93]	miR-361-3p/STX17	Promote	ARPE-19, Y79, SO-RB50, and WERI-RB1	Choroidal invasion and optic nerve invasion
circRNF20 [94]	miR-132-3p/PAX6 axis	Promote	ARPE-19, Y79, SO-RB50, and WERI-RB-1	TNM stage; worse overall survival rate

**Table 4:** Pathologic phenotypes of retinoblastoma.

	HW Rosettes	FW Rosettes
Degree of differentiation	Well differentiated	Poorly differentiated
Lumen	Lack of a distinct lumen	Distinct
